# Open Defecation Practices in Lodwar, Kenya: A Mixed-Methods Research

**DOI:** 10.1177/1178630219828370

**Published:** 2019-02-19

**Authors:** Phylis Jepkorir Busienei, George Morara Ogendi, Millicent A Mokua

**Affiliations:** 1 Department of Environmental Science, Egerton University, Egerton, Kenya; 2 Dryland Research Training and Ecotourism Centre, Chemeron, Kenya

**Keywords:** open defecation, sanitation, sanitation access, improved sanitation

## Abstract

**Background:**

As of the year 2014, about 2.5 billion people globally lacked access to improved sanitation. The situation is even worse in the sub-Saharan African countries including Kenya. The practice of open defecation (OD) peaks beyond 72% of the population in Turkana County, Kenya, despite various interventions to end it.

**Methods:**

This article reports on both qualitative and quantitative aspects of a cross-sectional study. A partially mixed sequential dominant (quantitative) status was used to understand various socioeconomic factors associated with OD practice in Lodwar’s human settlements, Turkana County. Simple random sampling technique was chosen to select participants for this study with the sample drawn from various administrative units of Lodwar. Standardized questionnaires, focus group discussions, and key informant interviews were used to collect data.

**Results:**

The quantitative findings revealed that culture was the leading factor as to why people practiced OD with a frequency of 44%. The findings further revealed that poverty was the major influencing factor for latrine ownership among the households (frequency 27%). Pearson χ^2^ tests revealed that there was a significant association between latrine presence and education level of the household head (χ^2^ = 107.317; *P* < .05), latrine sharing (χ^2^ = 403; *P* < .05), and occupation of the household head (χ^2^ = 74.51; *P* < .05). The quantitative findings showed that culture was by far the most common factor that contributed to the OD practice with a theme intensity of 31.1%. Further analyses identified 5 major cultural aspects that were associated with OD practice. This included sexual immorality, OD as a common habit, nomadic pastoralism, bride’s dignity and mixing of feces. Open defecation as a common habit among the respondents was the most cited factor that contributed to its rampant practice (theme intensity 31.3%) followed closely by nomadic pastoralism kind of life among the residents that limit latrine construction (theme intensity 28.1%).

**Conclusions:**

In addition to cultural aspects, high poverty levels influence latrine adoption and consequently OD practices. Future sanitation interventions addressing OD should assess and factor in these cultural aspects in such communities to come up with appropriate eradication measures which have otherwise been difficult to solve through poverty eradication and sanitation campaigns that have been in existence.

## Introduction

As of the year 2014, about 2.5 billion people in the world did not have access to improved sanitation with 1 billion practicing open defecation (OD).^1^ This is a major cause of millions of deaths from water-related diseases such as diarrhea among children under 5 years.^2^ Improved sanitation includes sanitation facilities that hygienically separate human excreta from human contact, whereas OD refers to the practice of defecating in fields, forests, bushes, bodies of water, or other open spaces.^1^ Open defecation is practiced in nearly all regions in the world. However, the practice is more common in India and some parts of the sub-Saharan Africa. In rural India alone, about 360 million do not have access to a toilet. However, over a third (37%) of the members of households still practice OD despite having a latrine facility.^3^

There is still inadequate access to improved sanitation facilities in sub-Saharan Africa with approximately 215 million people practicing OD as of the year 2013.^4^ Nonetheless, there was an improvement going by World Health Organization (WHO)^5^ 2015 report on “World Health Statistics” that shows increased use of improved sanitation in Africa from 25% in 1990 to 32% in 2013. This increase, however, consisted more of access to a simple pit latrine, which has deficient levels of privacy, hygiene, and safety. The situation is no different in Kenya. Roughly 50% of the population in rural areas lack access to a basic sanitation facility with 5.6 million Kenyans (14% of the total population) still practicing OD.^6^

Low-income levels have been found to be the major contributing factor to the problem of OD with individuals lacking access to a latrine facility spending 2.5 days or 60 hours per year searching for a place to defecate.^1^ A similar report by WSP,^7^ 2012 indicates that the poorest populations are more likely to practice OD as compared with the wealthiest populations. Kenya is not an exception with its poorest communities (including Turkana) practicing OD 270 times than the rich communities. This is due to limited funds to construct such facilities. Therefore, access to sanitation facilities is lower in the higher poverty gap index countries as compared with lower poverty gap index countries.

Due to poverty levels, latrine facilities may be constructed using poor construction materials such as mud or grass and are often in poor conditions (for instance, stagnant water or feces spread on the latrine floor). These practices may encourage OD practices. Construction of quality toilets may help reduce the OD practices. The recent emphasis on community participation in good sanitation programs such as the Slum Sanitation Program in Mumbai has pointed out that construction of toilets that meet the people’s needs is required to overcome the problem of OD.^8^ Low latrine coverage encourages long queues, especially in the morning, which in turn force these populations to practice OD.^8^ To achieve the sanitation target of the Sustainable Development Goals, the poor need to be helped to eradicate OD practice.^9,10^

How countries promote latrine construction and use is important in achieving Open Defecation Free (ODF) societies. Provision of subsidies for construction of these facilities has proven to be an effective health promotion strategy in some communities. A cross-sectional study in India, Indonesia, Mali, and Tanzania shows that households who were provided with subsidies to construct latrines showed greater odd of latrine usage than households who were encouraged to construct latrines through health promotions.^11^

Education level may also contribute to good sanitation and hygiene practices. The higher the level of education of an individual, the rational the mind of an individual and hence the wiser the person.^12^ Individuals who reached secondary and tertiary levels of education are aware of the negative impacts of OD and therefore tend to practice good sanitation practices. Most of the nongovernmental organizations today have constructed latrine facilities to the less-fortunate societies, but some do not even use these facilities. Participation of the family members in use of such facilities still lacks,^10,13^ and this is major because most of these individuals are not even aware of the importance of these facilities.

Weak or lack of sanitation laws and policies may lead to poor sanitation practices such as OD.^14^ In a qualitative research report from 8 countries, a larger percentage of the population agrees that the introduction of sanctions and strict rules to stop OD will reduce OD practice significantly.^15^ The introduction of sanctions in areas such as Lodwar may end OD practices.

A number of states, districts, or villages in various countries have fought against OD practice and have been declared ODF. In India, 152 535 villages, 85 districts across the country, Himachal Pradesh, Kerala, and Sikkim state have already been declared ODF under the Swachh Bharat Mission, the Center’s flagship program.^16^ However, fecal waste management still remains a great challenge, especially in poor and growing urban areas in many developing countries. A study in India showed that the lack of water could not explain rampant cases of OD as 90% of the population in rural India have access to improved water sources.^17^ A review study in rural Indonesia showed that sanitation interventions only have a small impact on latrine construction and utilization by communities.^18^ The Indian Government has provided subsidies for construction of latrines as one of the interventions to curb the practice of OD. However, this has yielded no fruit as the OD practices still persist in rural India despite India’s strong economic growth.^19^

There have been several interventions to end OD in Turkana such as Community-Led Total Sanitation (CLTS) introduced in 2007 and the ODF Rural Kenyan Campaign launched in the year 2011. These campaigns coupled with the expansion of sanitation facilities may not have been critical efforts to achieve meaningful health outcomes as OD cases are still rampant. Combining such efforts with cultural interventions may be an effective method for achieving ODF societies.^10,11^

Various studies have been done to assess factors that limit latrine adoption in various countries. However, these factors majorly focused on income and education levels. A study in rural India between 2005 and 2012 showed that education, economic status, and households’ demographic structures are weakly associated with latrine adoption.^20^ How cultural factors can be reshaped in communities that practice OD still remains largely unexplored. In Turkana, Kenya, there is limited research that has been done to exploit various factors associated with OD practice and especially cultural aspects. The OD practices peak beyond 72% of the population despite efforts to eradicate it.^6^ It is against this background that this study was conceived to assess various underlying socioeconomic factors that contribute to OD practices in Turkana County, Kenya.

## Methods

### Study setting

**Figure 1.**
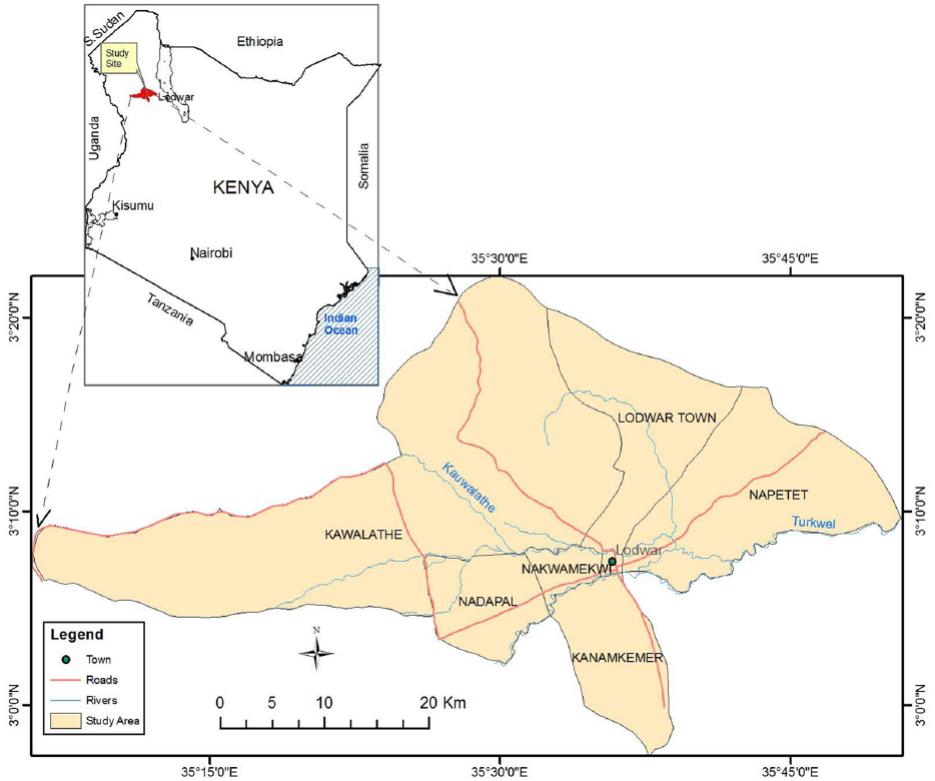
Map of the study area. Source: Topographic map of Kenya; scale 1:100 000—field survey.

The study was conducted in Lodwar town located within an Arid and Semi-Arid Land (ASAL) in Turkana County (Figure 1). Turkana County is situated in north-western Kenya and it borders Uganda, South Sudan, and Ethiopia. It is ranked the poorest County in Kenya with a poverty line of 94.3% (doubling the national rate of 45.9%) according to the international poverty line of US $1.90 a day. Lodwar town, which forms the study area, is the main headquarters of the County and it lies within the GPS coordinates 3° 07′ 8.80′′ North and 35° 35′ 17′′ East. The population living here cannot afford improved sanitation facilities such as VIP latrines, covered pit latrines, connection to a septic tank, or a sewer.^7^ They lead a nomadic pastoralist life, and only 18% of the population can read and write.^6^ The study was conducted in 4 administrative wards of Lodwar with a population of 41 120 households. This area was chosen due to rampant cases of OD reported in the region.

### Study design

This cross-sectional study exploring socioeconomic factors associated with the OD practices employed a partially mixed sequential dominant status design including quantitative and qualitative (thematic content analysis) components.^21^ The quantitative component was accorded more weight than the qualitative component in addressing the overarching research question. The qualitative component elaborated more on these factors thus giving a deeper meaning to the situation. A mixed-methods research provides a more balanced perspective by combining the benefits of both methods as well as offsetting the weaknesses that result from using one method alone.^22^

### Target population, sample size, and sampling

The target population for the quantitative study was adult household heads aged at least 18 years or their designated representatives, both who could give accurate data. The minimum sample size to assess the socioeconomic factors associated with OD practice was estimated $n={Ζ}^{2}\times Ρ(1-Ρ)/d^{2}$ using Kish formula for determining sample size for estimating population proportions, where *n* is the required sample size; *Z* is the statistic for a level of interval (at 95%, *Z* = 1.96); *P* is the population proportion, that is, 0.72 (the percentage of the population known to practice OD in the study area); and *d* is precision which is 0.05.

Using this formula, a minimum sample size of 310 households was estimated. Anticipating a 30% nonresponse rate, a final sample of 403 households was estimated.^23^

The total number of households in the study area was 41 120. Proportionate simple random sampling technique was employed to select 101 households from each stratum. Being the smallest stratum, 100 households were sampled in Kawalathe. Kanamkemer and Kawalathe settlements were the low middle-income areas, whereas Napetet and Nakwamekwi were the low-income settlements.

The target population for the qualitative component was the key informants and the focus groups. A total number of 20 key informants were selected based on their willingness and the fact that they have firsthand knowledge about the community.^24^ Most of these participants were from organizations that deal with water and sanitation issues, for instance, Lodwar Water and Sanitation Company, Save the Children, and National Environmental and Management Authority. In addition, 10 focus group discussions (FGDs) comprising 5 to 8 participants per group were selected purposely based on their willingness to participate in the study.^25^ For all the 4 administrative units, there were 3 female FGD groups and 7 male FGD groups. As the study targeted household heads, few female FGD groups were selected. This is because, in some countries including Kenya, the number of female-headed households is about one-third of the total households.^26^

### Research instruments

There were 4 research assistants in the study. The research assistants had obtained a bachelor’s degree in environmental science and had prior experience in data collection. The student researcher and the supervisor trained them for 5 days before data collection.

Before the actual study, a pilot study was done during the last 2 days of the training in Nadapal human settlements, and one of Lodwar towns with similar ecological conditions was selected to pretest the tools. A standardized questionnaire, whereby all the respondents were exposed to the same nature of questions and the same system of coding their responses, was used to collect quantitative data. The questionnaire contained 45 closed-ended questions concerning the respondent’s personal details, fecal disposal practices, and the Knowledge, Attitude, and Practices (KAP) questions on household’s fecal management practices. Respondents were free to answer or not to answer any question they felt was inappropriate to answer. Due to high illiteracy levels in the study area, in-person interview procedure was employed to administer and retrieve the questionnaires as this was considered less burdensome to those respondents who could not write out their responses. It also provides a high response rate and an opportunity to observe the household sanitation conditions thus providing a room to fill the observation checklists. A total of 10 households here were sampled to collect quantitative data. Two FGDs with women and men (18-80 years) from both low- and high-income areas were conducted to elaborate more on quantitative data. Two key informant interviews (KIIs) were also conducted with one village elder and a community member.

Quantitative data collection took place from October to mid-December, 2017, using a standardized questionnaire and an observation checklist. Based on prior studies that focused on factors that contribute to OD, the independent variables included household head’s education level, income levels, religion, and cultural practices. The dependent variable was OD practice.^10,13,14,27–32^ Qualitative data collection took place in February 2018. Using an FGD protocol, 3 FGDs were done in Nakwamekwi, 2 in Napetet, 3 in Kanamkemer, and 2 in Kawalathe. Prior to the study, the respondents were notified and were all able to meet at the agreed place and time. All the 4 enumerators handled 1 FGD at a time. Refreshments were offered to the participants. Using a KII protocol, 20 KIIs were conducted majorly at offices of the selected participants as well as homes. Both the FGD and the KII questions were based on factors contributing to OD, nature of latrines, and major OD hotspots with the average time for both the KIIs and the FGDs being 1 hour. There were additional questions for female FGDs on challenges of latrine access. In both the KII and the FGDs, note-taking and audio-recording were employed to store data.

### Data management and analysis

The collected data on the questionnaires were coded then entered into an SPSS database. Quantitative data were then checked for completeness. Frequencies and valid percentages were employed to analyze descriptive data. Pearson χ^2^ tests were used to analyze data on the various socioeconomic factors that are associated with OD practice. After all the analyses had been done, quantitative data obtained were represented in the form of tables. All levels of significance were tested at α = .05.

For the qualitative data, once the FGDs and the KIIs were done, the audiotape of the discussions was carefully transcribed and others were translated. After the data had been transcribed, it was coded following keywords, key concepts, or reflections in vivo and analyzed for common themes to achieve improved organization when pulling out the results and the key findings. The codes were then read by more than one researcher to check the consistency of the codes. The name of each theme was finalized and its description was written and illustrated with some quotations from the original text to communicate its meaning better.

Major themes were recorded and computed as follows^33^:

$$\begin{array}{l} Theme\;Frequency=Number\;of\;participants\;who\;\\ \;\;\;\;\;\;\;\;\;\;\;\;\;\;\;\;\;\;\;\;\;\;\;\;\;\;\;\;\;\;\;\;\;\;\;\;\;\;\;\;\;\;\;\;\;\;\;mentioned\;a\;particular\;theme\\ Theme\;Intensity=\frac{\begin{array}{l} Number\;of\;responses\;referring\;\\ to\;a\;particular\;theme\times 100 \end{array}}{\mathrm{Total}\;\mathrm{number}\;\mathrm{of}\;\mathrm{responses}\;\mathrm{in}\;\mathrm{the}\;\mathrm{study}} \end{array}$$

### Ethical issues

A research permit from Egerton University Research and Ethics Committee as well as the National Council of Science, Technology and Innovation (NACOSTI/P/18/77199/25718) was obtained before the study. Further approval was sought from the community leaders in the study area and the local authorities before the study began. Just before administering the questionnaires and audio-recording the FGDs and the interviews, written informed consent was obtained from each participant.

## Results

### Characteristics of study participants

There was a 100% response rate in all the data collection tools. All the 403 household heads participated in the quantitative study, and this was mainly because it involved in-person interviews. All the questionnaires were retrieved. In the absence of a household representative, the enumerator was able to select another household. There were 206 responses captured in the qualitative interviews and were coded to form 5 major themes and 5 cultural themes. About 87% of the respondents identified themselves as Christians (Table 1).

**Table 1.** Characteristics of study participants.

Characteristic	No. (%)	Characteristic	No. (%)
Administrative unit		Family size	
Kanamkemer	170 (42)	0-4 members	137 (34)
Napetet	33 (8)	5-9 members	203 (50)
Nakwamekwi	140 (35)	10-14 members	57 (14)
Kawalathe	60 (15)	15 members and above	6 (2)
Gender		Occupation of H/head	
Male	151 (38)	Employed	53 (13)
Female	252 (62)	Unemployed	192 (4)
Age, y		Casual laborer	75 (19)
18-28	124 (31)	Business	83 (21)
29-39	152 (38)	H/Head’s education level	
40-50	76 (19)	Primary level	129 (32)
51-61	37 (9)	Secondary level	86 (21)
62-72	13 (3)	Tertiary colleges	36 (9)
73 and above	1 (0)	University	17 (4)
		Illiterate	135 (34)

### Quantitative findings

As shown in Table 2, 81% of the sampled households did not possess a latrine facility; 27% of the respondents attributed the lack of latrine to high poverty levels in the region; and 44% of the respondents attributed OD practices to the culture of the people in the area. A total of 20% of the respondents were scared of using a latrine with most of the reasons being loose sand used to construct the latrines. A total of 333 (83%) respondents stated that the latrine construction materials influenced latrine ownership; 62% of the respondents stated that they had received some advice and guidelines on the importance of latrines before (Table 2).

**Table 2.** Socioeconomic factors associated with OD.

Characteristic	Frequency	%
Latrine presence		
No	326	81
Yes	77	19
Factors associated with latrine ownership		
Poverty	110	27
Poverty and culture^a^	64	16
Loose sand	53	13
Poverty, culture, and law enforcement^a^	51	13
Culture	38	9
Poverty and education level^a^	31	8
Law enforcement	29	7
Education level	27	7
Why do people practice OD		
Culture	179	44
Sharing latrines, feces on the latrine floor, tattered latrine walls, and culture^a^	73	18
Sharing latrines, feces on the floor, almost filled-up latrines and tattered latrine walls^a^	42	10
Tattered latrine walls	37	9
Almost filled-up latrines	18	5
Sharing of latrine with many households	16	4
Feces present in the latrine floor	14	4
Leaking latrine roof and stagnant water on the floor^a^	9	2
Bad odor in the latrines	8	2
Presence of flies, sharing of latrine, culture, and feces on the latrine floor^a^	5	1
Presence of flies in the latrine	2	1
Scared of using a latrine		
No	321	80
Yes	82	20
Why scared of using a latrine		
N/A for those not scared	321	80
One can fall inside	61	15
For some, one has to pay to use them	10	3
One has to clean the latrine when it is dirty	6	2
Its maintenance is costly	5	1
Do construction material influence latrine use		
Yes	333	83
No	70	17
How do construction material influence latrine use		
Some are expensive	101	25
Some are expensive and some do not offer enough privacy^a^	65	16
NA for those thinking there is no influence	64	16
Some are expensive, some do not offer enough privacy and some have to be cleaned with water (limited)^a^	42	10
Some do not offer enough privacy	35	9
Some are poorly constructed and can collapse	28	7
For some, one has to clean it with water which is limited	27	7
Some are poorly constructed and some are expensive^a^	23	6
Hygiene advice received		
Use latrine for defecation, safe disposal of babies feces and wash off hands with soap after defecation^a^	248	62
None	67	17
Use latrine for defecation	24	6
Use latrine for defecation and safe disposal of babies feces^a^	30	7
Safe disposal of babies feces	20	5
Wash hands with soap after defecation	13	3

Abbreviation: OD: Open Defecation.

a Respondents who stated more than one response.

### The KAP relating to OD

About three-quarters (76%) of the respondents agreed that OD was unsafe, whereas 72% of the respondents agreed that latrine sharing was an unsafe practice (Table 3). About 70% of the respondents were aware that some illnesses were related to OD practices, whereas 49% of the respondents agreed that the OD practice had become part of their tradition.

**Table 3.** Presentation of Knowledge, Attitude and Perception (KAP) questions on OD.

Characteristic	Agree	Undecided	Disagree
	%	%	%
OD is unsafe	76	11	13
Latrine sharing is unsafe	72	15	13
Some of the illnesses are related to OD	70	15	15
Religion is against latrine	0	16	84
OD is a tradition	49	13	38
Flies encourage OD	11	7	82
Odor encourages OD	10	10	80
Tattered latrine walls encourage OD	86	12	5
Poor flooring materials encourage OD	84	122	5
Almost/filled-up latrines encourages OD	92	5	3
Feces on latrine floor encourages OD	87	10	3

Abbreviations: OD: Open Defecation.

Privacy was a major concern for most respondents, and 86% of these respondents agreed that tattered latrine walls and poor roofing materials encouraged OD practices. Safety was also a major concern for most respondents, and 84% of the respondents agreed that poor flooring material, for instance, loose sand, encouraged the OD practices. Finally, most respondents were concerned about the cleanliness of the latrine with 87% of the respondents agreeing that the presence of feces on the latrine floor encouraged OD practice with only 3% of the respondents strongly disagreeing with the statement (Table 3).

### Pearson χ^2^ tests of associations

Pearson χ^2^ tests were run to evaluate whether there was a significant association between the presence of latrines and the education level of the household head. Table 4 shows that at χ^2^ = 107.317; there was a significant association between the education level of the household head and latrine presence in the study area (*P* < .05). Those who attended school (primary, secondary, and tertiary levels) were more likely to own a latrine as compared with those who did not attend school (illiterate).

**Table 4.** Latrine presence as stratified by location, sharing, household head’s level of education, and occupation (n = 403).

Characteristic	Latrine presence		*P* value
	Yes (%)	No (%)	
Settlement			
Kanamkemer	19	81	χ^2^= 0.424, *P* > .05
Napetet	18	82	
Nakwamekwi	18	82	
Kawalathe	22	78	
Head’s occupation			
Employed	55	45	χ^2^= 0.424, *P* < .05
Unemployed	5	89	
Casual labor	20	80	
Business	29	71	
Head’s education level			
Primary	8	92	χ^2^= 107.317, *P* < .05
Secondary	37	63	
Tertiary college	53	47	
University	71	29	
Illiterate	3	97	
Latrine sharing			
Yes	65	0	χ^2^= 403.000, *P* < .05
No	35	0	

There was no significant association between latrine presence and the administrative units present in the study area (χ^2^ = 7.058, *P* > .05). However, Nakwamekwi settlements had a higher number of the population without access to a latrine facility (82%; Table 4). There were 2 internally displaced person (IDP) camps in Kanamkemer settlements, one (Kanan IDP camp) of which has a total number of 610 households with only 6 toilets. Nakwamekwi settlements also had 2 refugee camps, one (Nataparkakono IDP camp) of which had 146 households that had only 1 latrine that was completely filled up. Nakwamekwi IDP camp had no latrine facility, and the residents here used bushes and thickets near the river for defecation.

There was a significant association between the total number of latrines identified (77 latrines) and latrine sharing (χ^2^ = 403, *P* < .05) (Table 4). A total number of 50 latrines were being shared. The shared latrines were mostly pit latrines, and some of them were constructed by the government as well as the joint community.

There was a strong association between the occupation of the household head and the presence of a latrine in the sampled households (χ2 = 74.51, *P* < .05; Table 4). The higher the number of household heads employed, the higher was likely to possess a latrine facility (55%) as compared with household heads who were unemployed (5% with latrine).

### Qualitative data on socioeconomic factors associated with OD

Due to low-income levels, latrine sharing was a common practice in the study area with more than 16 households using a single latrine. In one of the interviews with the village elder in Kanan IDP camp, he stated,

. . . the residents in this IDP camp especially adults face major challenges when it comes to latrine access, and they have to wake up very early in the morning to go and relieve themselves in the open or wait until late in the evening. The nearest bushes are commonly used as well as the three excavations that were left open during the construction of this IDP camp. (Personal Communication, 2018)

**Figure 2.**
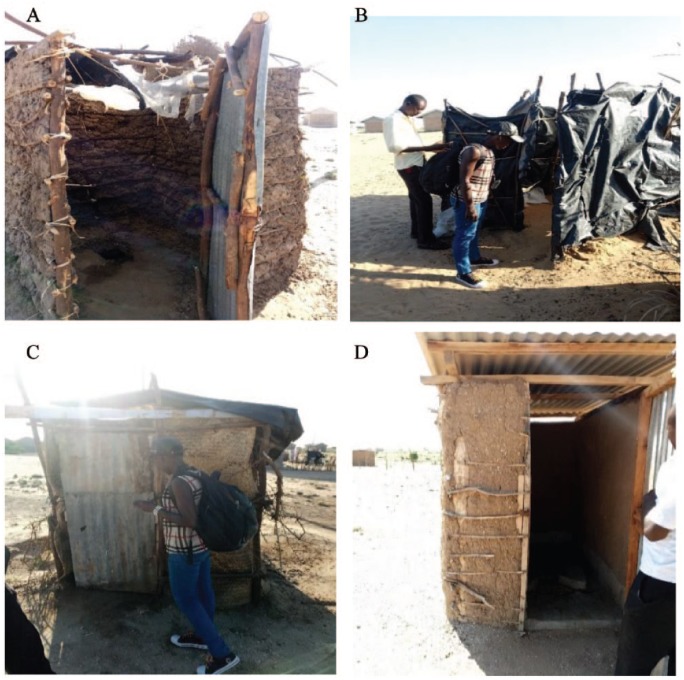
Figure A,B,C and D Showing the nature of latrine facilities in the study area.

The few available latrines were constructed using poor materials as 48% of the population were not employed (Figure 2). The village elder added,

I know of four latrines that have collapsed in this area. One of them collapsed and injured one user but was rescued by the family members. This has so far scared many users, and residents here prefer going to the bush to defecate. For those who have a latrine facility, most of them are constructed using poor quality timber that rots even in one year and may collapse killing some of the users. (Personal Communication, 2018)

Daily habits coupled with low-income levels were also a significant factor that encouraged the OD practice. In one of the male FGDs in Kawalathe settlements, a respondent stated,

. . . We are used to using these bushes along the river to relieve ourselves from when we were born. The sand is very loose too, and the latrine can collapse, and so I am more comfortable using the bush than the latrine…the bush is near and convenient, it also provides enough privacy compared to a latrine that I would have constructed using grass material. (Personal Communication, 2018).

In another female FGD in Kanan IDP, one respondent stated,

. . . Even if we are provided with a latrine facility today, I am sure that the OD practice will still be present since we are brought up in a society where the practice is very common. For instance, I cannot use the same latrine with my father-in-law. OD has become a habit for most of us. If we are used to going to the bush, we will still go to the bush even if we are provided with a toilet. (Personal Communication, 2018)

Lack of strict laws that govern OD practices was also stated as one factor contributing to rampant OD cases. In 2 KIIs with the officials of Lodwar Water and Sanitation Co. Ltd (LOWASCO) and Save the Children, respectively, the interviewees stated that the major reason why the residents used bushes and thickets in the stadium as well as the arboretum to defecate was major because there are no strict laws that prohibit residents from doing so. One interviewee added up that some of the residents were in a position to construct a latrine but cannot do so because there is no law to enforce such a practice. In another KII with a staff of Water, Sanitation and Hygiene (WASH) in Save the Children, he stated,

. . . even if the residents are provided with enough latrine facilities in the town today, people will still use the arboretum as well as the stadium, not even because it has become a habit but because there are no rules that prohibit OD practice. People like being monitored and with an introduction of a sanction, I am sure the OD practice in these two sites will be eliminated. (Personal Communication, 2018)

All the 10 FGDs and 20 KII guides were analyzed for common themes on the socioeconomic factors associated with OD practices. The in vivo (the use of respondent’s exact words) and descriptive (coding of major themes) coding analyses identified 5 major themes emerging from respondents on various socioeconomic factors associated with OD practice: *culture, poverty, limited laws, low education*, and *loose sand*. The 5 themes referred to various socioeconomic factors associated with OD. As shown in Table 5, the results of the quantitative data owed that culture of the communities was significantly contributing to OD practice in the study area (theme intensity 31.1%) as compared with poor sand that limits latrine construction thus encouraging OD practice.

**Table 5.** Frequency of themes describing various socioeconomic factors associated with open defecation.

Themes/response	FGDs	KIIs	n	Theme intensity (%)	Culture themes/response	FGDs	KIIs	n	Theme intensity (%)
Culture	47	17	64	31.1	Habit	15	5	20	31.3
Poverty	30	18	48	23.3	Pastoralism	15	3	18	28.1
Limited laws	25	12	37	18.0	Preserve dignity	7	4	11	17.2
Low education	17	13	30	14.5	Sexual immorality	5	3	8	12.5
Loose sand	22	5	27	13.1	Mixing of feces	6	1	7	10.9
**Total**			**206**	**100**	**Total**			**64**	**100**

Abbreviations: FGDs, Focus Group Discussions; KIIs, Key Informant Interviews.

Adapted from Wao et al.^33^

Further analyses were conducted on how culture influences OD practice and 5 major themes were identified: *habit* to mean that the community members were used to defecating in the open, *pastoralism* to mean that nomadic pastoralism kind of life limited latrine construction, *bride’s dignity* to mean that latrines were only constructed during the welcoming of the pride to preserve the dignity of the family, *sexual immorality* to mean that men and women using the same toilet was considered as a form of sexual immorality, and *mixing of feces* to mean that using the same toilet meant mixing of feces which is considered as impure. The results of the meta-analysis revealed that OD was practiced because it had become a habit (theme intensity 31.3%) and the communities were used to it as compared with the mixing of feces which was considered as impure (Table 5).

## Discussion

Access to improved sanitation is an important aspect of public health.^5^ Using a mixed-methods research approach, this study aimed at identifying various socioeconomic factors associated with the practice of OD. Five major socioeconomic factors were identified: *culture, poverty, limited sanitation laws, low levels of education*, and *loose sand*. Further analyses identified 5 major cultural themes that were associated with OD and included *OD as a common habit, nomadic pastoralism, bride’s dignity, sexual immorality*, and *mixing of feces*.

First, poverty was one important factor that influences latrine ownership as well as OD practices in this study. Employment goes hand in hand with increased earnings, good health, as well as other socioeconomic outcomes. There was little latrine coverage in households with low-income sources as compared with households with high-income sources in the study area with the most of the respondents stating that construction materials (perceived as being expensive) influenced latrine ownership. In a similar study to assess factors related to OD behavior among school-age children in West Lombok, Indonesia, most of the respondents with low-income levels did not have a latrine facility at their homes as they cannot afford the cost of construction.^13^ According to KNBS and SID report, 2013, only 6% of the population in Turkana County works for pay and is ranked the last and is the poorest county in Kenya.^6^ Households with low-income levels will often place a lower priority on sanitation.^34^

People living in low socioeconomic status cannot afford improved sanitation and thus are less likely to spend on sanitation.^35,36^ A cross-sectional study from 2008 to 2012 from households in rural areas of Tanzania, Indonesia (East Java), and multiple states of India reported that more than 60% of the households living in low socioeconomic status practice OD compared with less than 1% of the households living under high socioeconomic status.^28^ This is majorly due to the cost of latrine construction as reported by 83% of the respondents in this study. A similar study in Ethiopia shows that in households with an annual income of US $300 or more per year, latrine ownership increased by 2-fold as compared with households with less than US $300 per year.^37^ Another study to assess patterns and determinants of latrine use in Odisha, India, however, suggests that the construction of latrines by the government alone was insufficient to address the practice of OD^38^ adequately.

Low-income levels lead to the use of poor latrine construction materials which do not offer enough privacy. This may encourage OD practices. The study area is also characterized by loose sand that requires good constructed latrines. This was a major problem as most of the household heads were unemployed. In this study, respondents preferred going to the bush than using a latrine that had its walls tattered. A similar study to assess factors influencing OD and latrine ownership in Cambodia, India (Rajasthan, Meghalaya, and Bihar), Indonesia (East Java), Kenya, Malawi, Peru, Tanzania, and Uganda points out that it is very important to have a latrine with all its walls enclosed as latrine privacy is a crucial factor.^15^ This is especially for women as most of them do not like exposing their body parts and is a motivation why people construct latrines rather than defecating in the open.

Low-income levels may also encourage latrine sharing which was a common practice in the study area with half of the latrines being shared by more than one household. Latrine sharing goes hand in hand with latrine filthiness.^39^ Latrine filthiness may have been one of the factors why some households possessed a latrine but was not using it with more than three-quarters of the respondents agreeing that human feces on the latrine floors and filled/almost filled-up latrines encouraged the practice of OD. A formative study to examine who is likely to own a latrine in 2008 and 2012 from households in rural areas of Tanzania, Indonesia (East Java), and multiple states of India also reported that the perception of the latrine users toward the use of dirty latrines is negative and thus they may not want to use an unhygienic facility and may opt for OD.^28^

Second, these study findings show that latrine ownership in the study area was largely associated with the respondents’ levels of education. Household respondents who did not own a latrine were mostly illiterate and those who had primary levels of education. The education level of a household head is an important aspect toward human development as it exposes him or her to various opportunities as well as increased earnings. A similar study to assess factors that facilitate latrine adoption in Tanzania reported that education was significantly associated with OD. Respondents who had reported to have attended school had 5.26 greater odds of using a latrine facility as compared with those who had never attended school.^32^ Educational status of mother and the presence of secondary school student are the leading factors to latrine use and consequently the practice of OD.^40^

This study also identified limited or absence of strict laws govern the sanitation practices as the third factor that contributes to OD practices in Lodwar. Individuals who have a perception that the presence of village rules and regulations in place that inhibit the OD practice have greater odds of owning a latrine.^28^ However, the development, implementation, and monitoring of sanitation laws and policies require adequate budget allocations^4^ which is a major problem in most developing countries. A similar study to assess the elimination of OD and improved sanitation in Nepal reported that presence of sanitation regulations was one of the social pressures that drove households to adoption and sustained use of latrines.^41^

There were households, however, that possessed latrine facilities in the study area but were not using them simply because according to them, latrine ownership was a necessary requirement. A similar report from Kajiado, Kenya, shows that some of the households in the region possessed a latrine facility but were not using it because they are not used to defecating in the latrines. The owners reported that those latrines were only constructed for the health officials and the government who forced them to do so.^42^ These communities tend to have deep-rooted values on such practices. Findings from a similar study among school-age children in West Lombok, Indonesia, reported that such communities are often comfortable defecating in the open even if such situations are uncertain.^13^ As a result, such communities are not too oriented to any form of regulations. A combination of fines, shaming, and withholding of community benefits may be considered as successful sanitation elements that may promote latrine construction as well as its usage among such communities.^15,43^

Various sanitation campaigns have been conducted in Turkana County with most of the respondents being fully aware of such advice as the use of latrine for defecation, safe disposal of children feces, and washing of hands after defecation. However, OD is still a challenge in the region with respondents citing OD as a cultural habit that has been in existence over a long period of time. A report by World Bank to assess whether sanitation campaigns get people to use toilets in Tanzania showed that sanitation campaigns reduced regular OD but occasional OD continued.^44^ Even with the provision of infrastructure to construct latrines, the presence of nearby water, habits, sanitation rituals, and daily routines are some of the factors that contribute into little latrine adoption.^31^

Findings from this study strongly associate OD practice with cultural habits as the fourth socioeconomic factor. Daily habits determine the health conditions of a population. Often, several factors play a role in influencing the formation of these habits.^31^ The process to change these habits is often hard if the habits have been internalized and embedded in the everyday life of such populations. A similar study to assess the effects of India’s Total Sanitation Campaign on defecation behaviors in rural Madhya Pradesh reported that changing social norms and behaviors achieved modest reductions in OD cases.^45^ This is a field which has not been looked at in depth in Lodwar, Kenya. Even with the presence of a latrine facility, some of the households do not use these facilities. Such compounds were characterized by the presence of feces scattered over the compound.

Various cultural aspects played a role in influencing OD practices in the study area with OD as a daily habit/routine being the most cited aspect that contributed to OD practice. A cultural value which has been learned from childhood is often a difficult thing to change as mothers train their children to defecate in the open and later on in life it becomes a habit.^31^ So even with changes in sanitation practices, such communities may not change what they are used to. These findings are similar to those of a study that assessed sociocultural and behavioral factors constraining latrine adoption in rural coastal Odisha with the men respondents known to practice OD reporting that latrines were suitable for women only, who were home most of the time, and especially a newly-wed daughter-in-law.^31^

Owing to the occupation of such populations, men, especially farmers, cannot come back home to access a latrine and can defecate anywhere. Findings from a similar study in Uttarakhand, India, reported that wealthier villagers could afford to construct latrines, but OD practice was considered more convenient to them especially when practicing agriculture or transhumance.^29^ Complementarily, 60.4% of the respondents who were known not to use latrines in Denbia district, Northwest Ethiopia, attributed latrine use to long-live habit with 18.9% considering OD a comfortable practice.^40^

Pastoralism kind of life was also cited as the second leading cultural factor that hindered most households from constructing a latrine. Most of the counties in Kenya with high OD rates have a large proportion of pastoralists who practice livestock keeping.^35^ These nomadic communities tend to move with their animals in search of water and pasture and rarely carry mobile toilets along with them. They perceived latrine construction as wastage of funds as they were not going to stay in one location anyway and would rather defecate in the open. Findings from a similar study in rural Tanzania shows that livestock-keeping was significantly associated with OD practice with 15 (16%) of the households practicing OD earning their income through livestock-keeping.^32^

Dignity and sexual immorality were also some of the cultural aspects to OD practice. Households were only likely to construct a latrine during the welcoming of the bride to her new house and defecating outside was perceived to lower the prestige of the family. Some of the respondents also stated that having one latrine in a compound that is shared among all the members of the households was considered as a form of sexual immorality. Relatives, for instance, a father and his daughter-in-law, are not allowed to use the same toilet as this is considered immoral behavior. Findings from a similar formative cross-sectional study from households in rural areas of Tanzania, Indonesia (East Java), and multiple states of India point out that cultural norms, such as the belief that male in-laws and females should not share the same latrine facilities, are associated with OD practice.^28^

Finally, the findings from this study indicated that using the same toilet among all the family members meant mixing of feces which is considered impure according to their beliefs. Similar evidence from a household survey in rural north India indicates that some percentage of the population continue to defecate in the open despite having a latrine facility. Such population believes that defecating in the open is healthier than using a latrine.^17^

### The novelty of this study

Existing interventions to end OD practices in Turkana County have been largely unsuccessful. This study highlights poverty, low levels of educations, limited sanitation laws and policies, loose sand, and culture as some of the leading factors that have contributed to rampant cases of OD in Lodwar, Kenya. Provision of infrastructure to construct latrines and awareness campaigns on the importance of good sanitation practices has majorly been some of the interventions to end OD in the study area. However, these efforts have not yielded fruits as there are significant and culturally engrained cultural barriers to latrine use as well as OD practice. Various cultural aspects have been pointed out in this study, and this presents a significant gap that other studies in Kenya have not looked at it much deeper. An assessment of these cultural aspects in such communities proves to be an appropriate method in understanding the reasons for rampant cases of OD, which may otherwise be difficult to solve through the provision of subsidies to construct latrines and sanitation campaigns that have been in existence.

### Study limitations

First, this study is only generalizable to the peri-urban population of 4 settlements in Lodwar and does not include the rural populations. Second, this study did not analyze the association of broader factors such as law enforcement or macro-social influence and OD practice.

## Conclusions

Results show that there is inadequate access to latrine facilities in the study area. Although poverty is a major factor that contributes to OD practice in quantitative studies, most of the respondents from the quantitative study revealed that culture is the most dominant factor why populations practice OD. The practice of OD has been inherited through generations who are known to practice it. The study concludes that even though poverty levels are high in the study area, provision of a latrine facility alone may not be able to solve the current issue of OD. Future sanitation interventions addressing OD should factor in these cultural aspects in such communities to come up with appropriate OD eradication measures which have otherwise been difficult to solve through poverty eradication and sanitation campaigns that have been in existence.
